# Selective release of the digital extensor hood to reduce intrinsic tightness in tetraplegia

**DOI:** 10.3109/2000656X.2011.558729

**Published:** 2011-04-19

**Authors:** Carina Reinholdt, Jan Fridén

**Affiliations:** National Center of Reconstructive Hand Surgery in Tetraplegia, Department of Hand Surgery, Sahlgrenska University Hospital, Göteborg, Sweden

**Keywords:** Surgical release, intrinsic tightness, tetraplegia, spasticity, finger extension

## Abstract

Patients with tetraplegia may have various degrees of spasticity in the hand ranging from a completely clenched fist to reduced control of grip at triggered spasticity. The objective of the present study was to evaluate the functional effect of the distal ulnar intrinsic release procedure to reduce intrinsic tightness. Seventeen patients with tetraplegia (37 fingers) and with prominent intrinsic tightness were operated on for distal intrinsic release with a modification of the procedure to include only the ulnar side of the proximal phalanx. All the patients had more pronounced tightness on the ulnar than on the radial side of the affected finger. Long fingers were consistently the most affected digits. The intrinsic tightness was released completely in all patients and the range of motion (ROM) was improved by 25%, and up to 45% in mild and severe cases, respectively. The good immediate effects of treatment as shown by increased ROM remained intact by 6 months postoperatively. These data suggest that the distal ulnar intrinsic release procedure is a simple and valuable way of reducing intrinsic tightness and improving hand function and grip for patients with intrinsic tightness. This procedure can be added to other procedures such as lengthening and transfer of tendons.

## Introduction

Patients with incomplete tetraplegia often suffer from spasticity in the hand varying from a completely clenched fist to decreased grip control at triggered spasticity. Some of them have intrinsic tightness due to the spinal cord injury (SCI) and subsequent spasticity. Other causes of intrinsic tightness may be a longstanding oedema or haematoma, trauma, CNS lesions, ischaemia, etc [1]. The interossei and the lumbrical muscles, the intrinsic musculature, act on the hand to flex the metacarpophalangeal (MCP) joints and to extend the interphalangeal (IP) joints [1]. When the intrinsic muscles are spastic or too tight, the gripping ability is affected. To flex the finger joints and to grip a tool may be difficult. In more severe cases with lack of extension function and severe spasticity, the extension of the MCP joints is limited and the opening of the hand is largely reduced. Naidu and Heppenstall stated that the MCP joint is unaffected in mild cases with intrinsic tightness and inability to flex the proximal interphalangeal (PIP) and distal interphalangeal (DIP) joints is the main complaint. In severe cases the MCP joints are involved with the consequential inability to grasp [2]. Harris and Riordan introduced an operation to release intrinsic tightness: distal intrinsic release [3]. The procedure has also been described by Lee and Gellman [4]. This procedure includes release on both the ulnar and radial sides of the extensor hood. However, we know of no studies on the use of this procedure in patients with tetraplegia and spasticity. The ratio complete SCI:incomplete SCI has changed over the past years as a result of safer cars, better emergency trauma care, etc [5]. The percentage of patients with incomplete tetraplegia is 55% of all patients with tetraplegia. These patients are often spastic, and many different conservative treatments may be used to reduce the problems with various results. Patients with spasticity resulting from spinal cord injury often present intrinsic tightness or spasticity, which is more prominent on the ulnar side. A logical consequence therefore is to release only the ulnar side, and to introduce the procedure in patients with spasticity.

## Patients and methods

**Figure 1:**
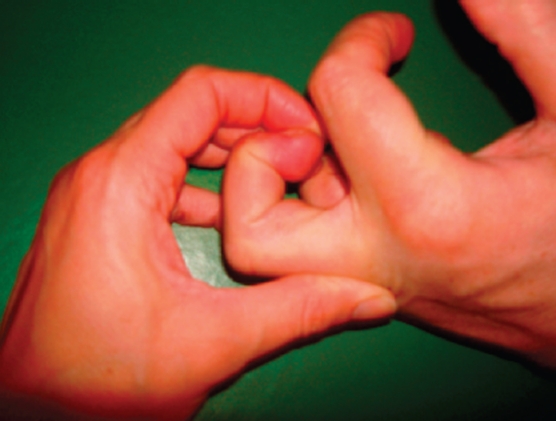
Bunnell's intrinsic tightness test involves passively holding the patient's MCP joint extended, and then passively flexing the PIP joint. There is intrinsic tightness if the PIP joint is difficult to flex.

The diagnosis of intrinsic tightness was verified by using Bunnell's test [6], which has also been described by Finochietto (Figure 1) [7]. In addition, a test was done to distinguish between intrinsic tightness on the ulnar and the radial sides. The radial and ulnar interosseus insertions were tested with the MCP joint in a maximally-deviated position to the radial and to the ulnar side, respectively and the tightness test was done on both sides. The passive range of motion of the MCP and the PIP joints were also measured. The patients were all tetraplegic and reconstructive hand surgery was planned including distal intrinsic release as a single procedure, and multiple procedures such as reconstruction of grip with tendon transfers and lengthening. All patients had previously tried conservative treatment of spasticity with unsatisfactory results. The patients were divided into 2 groups: mild and severe, depending on the degree of involvement of the MCP joints according to Naidu and Heppenstall [2]. The patients in the mild group had focal spasticity, and those in the severe group had spasticity in both the deep and superficial flexor muscles and a clenched fist. In the severe cases, the intrinsic tightness was hidden behind the spastic superficial and deep flexor muscles. When the spastic extrinsic muscles were lengthened or transferred, the intrinsic tightness became obvious, and was corrected with a distal ulnar intrinsic release.

**Figure 2:**
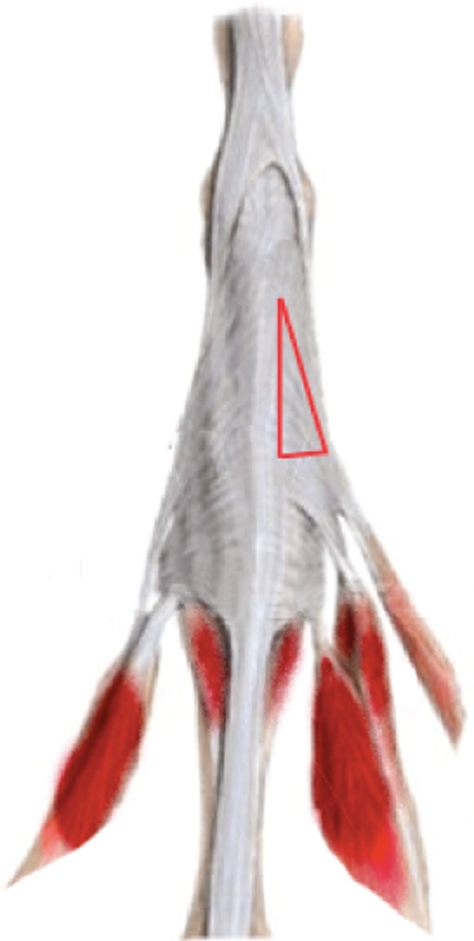
The extensor apparatus of a finger. The red triangle shows the area that should be resected in the distal ulnar intrinsic release procedure. The triangular piece consists of oblique fibres and the ulnar lateral band.

**Figure 3:**
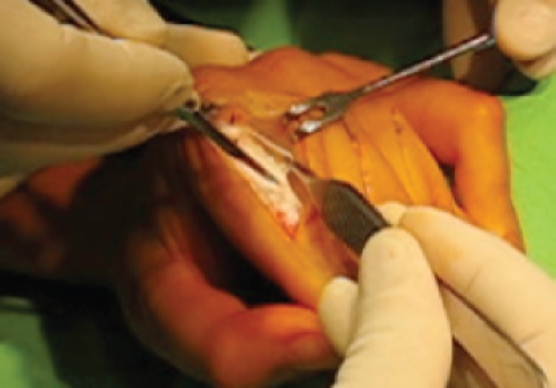
Distal ulnar intrinsic release. An oblique dorsal incision on the proximal phalanx exposes the extensor hood apparatus on the ulnar side and a triangular piece is resected. The triangle is roughly 5 mm at the base, 12–15 mm at the side, and the hypotenuse is 15–16 mm.

The patients were operated on with distal intrinsic release, when the oblique fibres of the extensor hood are excised [3,4]. The original procedure was modified to include only the ulnar side of the dorsal aponeurosis. The extensor mechanism is exposed through a dorsal oblique incision on the proximal phalanx. The ulnar side of the aponeurosis is identified and a triangular piece containing the lateral band and the oblique fibres is resected (Figures 2, 3). Release is sufficient when the intrinsic tightness as described by Bunnell's intrinsic tightness test is gone.

Postoperative pain control consisted of paracetamol and low-dose opioids, and in some cases NSAID. Antibiotics were not given routinely in planned reconstructive hand surgery. Follow-up and postoperative active training were the same in both the mild and severe groups. Measurements were taken preoperatively, peroperatively, and at follow-up at 1, 3, and 6 months postoperatively. Active motion was encouraged the day after the operation. A resting splint with the MCP and the PIP joints extended, or almost extended (depending on the simultaneous procedures) was used between the multiple training sessions 4 weeks postoperatively. At 4 weeks, the training changed to task-oriented exercises, such as daily living activities.

### Statistical analysis

**Figure 4:**
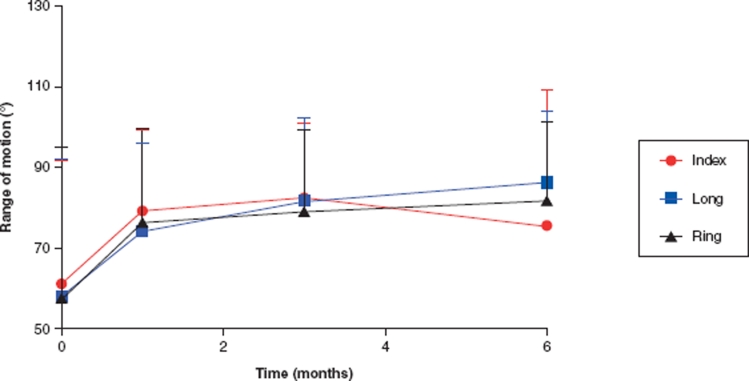
Mean (SD) results over time at follow-up at 1, 3, and 6 months. The biggest improvement of range of motion was during the first month after the operation.

Data are presented as mean ± SD. In addition to the metadata from each specimen for which only descriptive statistics were calculated (Figure 4), range of motion for all fingers in the PIP joint was analysed across the two groups (mild and severe). These data were screened for normality, and skewed to justify the use of a parametric one-way analysis of variance (ANOVA) with severity serving as the two repeated measures. For the individual fingers (index, long, and ring fingers), the Mann-Whitney test was used to assess the final effect of treatment (6 months) within the two severity groups. Significance level (α) was set to 0.05 for all statistical tests.

## Results

All the fingers that we operated on had intrinsic tightness, which was more prominent on the ulnar side. The middle finger was involved in all but one case. One patient had bilateral procedures in separate sessions. The level of injury was mainly C5-C7 and the patients were classified as IC OCu 3–6 and OCu X [8]. The mean age of the patients at time of operation was 48 years (range 23–75) and the mean time from injury to operation was 5 years (range 0.8–16). The mild group comprised 7 patients (14 fingers) and the severe group 10 patients (23 fingers). There were 6 women and 11 men.

All patients managed to initiate immediate postoperative active training according to the protocol. None of the patients developed excessive oedema, infection, or pain of a magnitude that it inhibited full participation in the postoperative exercises. On discharge from hospital 3 days postoperatively, all patients reported subjectively satisfactory results of the obtained and increased range of motion.

**Figure 5:**
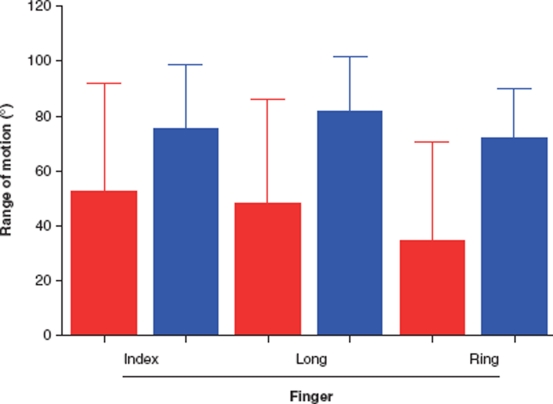
Mean (SD) passive range of motion of the PIP joints preoperatively compared with 6 months postoperatively in the severe group. There were significant increases in range of motion postoperatively in the long and ring finger. Red = before, and blue = after, operation.

**Figure 6:**
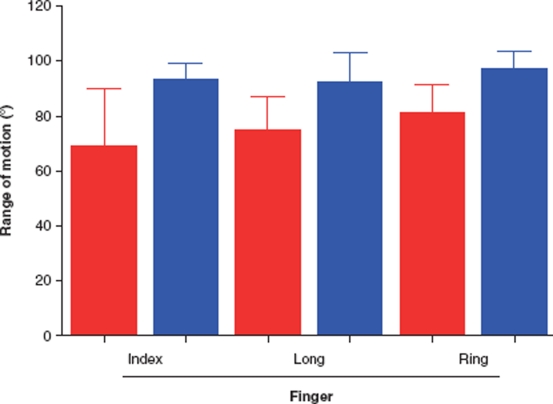
Mean (SD) passive range of motion of the PIP joints preoperatively compared with 6 months postoperatively in the mild group. There was a significant increase in the range of motion for the long finger. Red = before, and blue = after, operation.

At follow-up 1, 3, and 6 months postoperatively, range of motion for all fingers was maintained at the immediate postoperative level or slightly increased (Figure 4). All patients logged their training sessions and the use of the splint according to the protocol. At 6 months follow-up, those with severe tightness responded with significant increases in the range of motion postoperatively for the long (*p* = 0.019) and ring (*p* = 0.037) fingers (Figure 5). The increases in the range of motion (ROM) were 35° and 45° for the long and ring fingers, respectively. In the mild cases, a significant difference was found for the long finger; it increased its ROM from 65° to 90° (*p* < 0.05, Figure 6).

**Figure 7:**
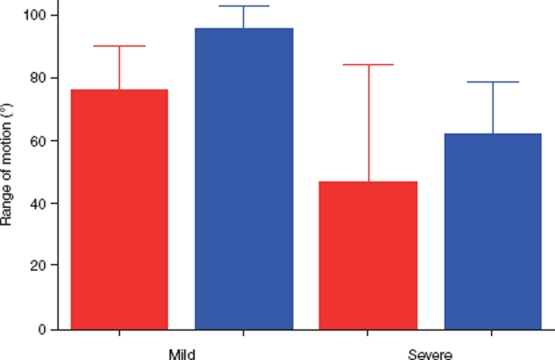
The patients in the mild group had a better range of motion at 1 month follow-up than the severe group, who had more operations. The severe group kept improving their range of motion during the following months. Red = before, and blue = after, operation.

The immediate effect of release differed, depending on the severity of the deformity, so in the mild cases there was a more pronounced immediate effect at 1 month compared with the severe cases. The patients in the mild group had better ROM preoperatively and maintained a good result. The patients in the severe group needed longer time to improve their ROM (Figure 7).

## Discussion

We have shown that the distal ulnar intrinsic release procedure reduces intrinsic tightness, and is a simple and valuable procedure for patients with tetraplegia and intrinsic tightness. In mild cases, in whom the PIP joint is involved exclusively, only the oblique fibres need to be resected according to Naidu and Heppenstall [2]. Extensive release of the dorsal aponeurosis is necessary when the MCP joints are involved, together with secondary contracture. In this study a triangular piece of the ulnar side of the dorsal aponeurosis was resected until the intrinsic tightness was sufficiently released, which is assessed subjectively by evaluating the resistance to flexion of the PIP joint while the MCP joint is held extended. It is easy to evaluate the release, because the result is instant (flexion >90°), which is a great benefit for the surgeon. Espiritu et al. showed that a minimum of 65% of the oblique fibres must be resected on the middle finger for adequate flexion of the PIP and fewer fibres on the other fingers [9].

The miscellaneous group of patients consisted of mild cases, where the main complaint was, for example, difficulties in holding slim tools, and more severe cases who had a clenched hand and inability to grasp, and where tendons were both transferred and lengthened at the same time. The patients in the mild group were instantly better and satisfied with better flexion and control of grip. The more severe cases, who had multiple simultaneous procedures and in whom intrinsic tightness was hidden in the spastic hand, were more difficult to evaluate because the function of the hand was dramatically improved by many reconstructions. However, it is likely that these patients would have been subjects for distal intrinsic release later if it had not been done straight away. The patients in the severe group needed longer time to improve (roughly 3 months) compared with the mild group who were doing well at 1 month.

The intrinsic tightness was released promptly when only a triangular piece of the dorsal aponeurosis was resected including the oblique fibres on the ulnar side. The patients had more ulnar intrinsic tightness than radial tightness. On the radial side of the proximal phalanx the radial interosseus insertions tend to insert more into bone, and the ulnar insert more into the aponeurosis [1]. Patients with incomplete spinal cord injuries and some residual function left in the interossei would hypothetically benefit from keeping the radial insertion intact to give them a strong key pinch. As most tetraplegic patients lack function of the first interossei, radial stability of the index finger is important as a target for the key pinch. By resecting only an ulnar piece, the radial tightness function may be restored together with release of the tight ulnar side to achieve a better-controlled grip.

In the more severe cases, in whom the intrinsic tightness was hidden by extrinsic flexor spasticity, tendons were lengthened and transferred simultaneously. In these cases the newly-discovered intrinsic tightness may result from either spasticity or tight lumbricals. A lumbrical plus finger can occur after lengthening of the FDP tendons [1]. The lumbricals are tiny in comparison to the interossei [10].

Releasing intrinsic tightness result in a better gripping ability and better grip-control for the patient. Preoperatively COPM (Canadian Occupational Performance Measurement) was made on 10 patients and showed an increase of 3.9 scale steps on performance and an increase of 4.5 on satisfaction. These data show that the procedure is both useful and satisfactory.

The middle finger was affected in all but one of the patients, possibly because the middle finger is powered by two dorsal interossei and these insert into the lateral band in a much higher degree than the 1^st^ and the 4^th^ interossei, which insert more often to bone [1]. Eladoumikdachi et al. showed that all the third dorsal interossei had insertions on the lateral bands, compared with 14% of the first, and 79% of the second, dorsal interossei [11]. Perhaps this more elastic insertion allows the spasticity to result in a tighter deformity?

The patients started with active exercises on the first postoperative day and this is thought be one reason why the patients maintain their ROM. This regimen prevents adhesions in the area of the wound. At 4 weeks postoperatively most patients had already achieved a good ROM. This can be compared with common postoperative treatment, which involves a cast for 4 weeks.

We acknowledge that this study is limited because of the small number of patients and the variability among the patients. However, the results show that this modified procedure is beneficial for the patients. Distal ulnar intrinsic release is now an established operation offered to patients with tetraplegia and spasticity in our unit. The procedure is technically easy, and sufficient release can be evaluated instantly.
